# Home monitoring of fetal heart rhythm: Lived experiences of women with anti-SSA/Ro52 autoantibodies and their co-parents

**DOI:** 10.1177/09612033241244465

**Published:** 2024-04-03

**Authors:** Joanna Tingström, Elin Öst, Gunnar Bergman, Åsa Burström

**Affiliations:** 1 Department of Obstetrics and Gynecology, 27106 Department of Clinical Science and Education Karolinska Institutet, Stockholm, Sweden; 2 Department of Pediatric Surgery, 59562 Karolinska University Hospital, Stockholm, Sweden; 3 Department of Women’s and Children’s Health, 27106 Karolinska Institutet, Stockholm, Sweden; 4 Department of Women’s and Children’s Health, Karolinska Institutet and Department of Pediatric Cardiolgoy, 59562 Karolinska University Hospital, Stockholm, Sweden; 5 Neurobiology, Care Science and Society, 27106 Karolinska Institutet, Stockholm, Sweden

**Keywords:** Pregnancy, congenital heart block, Doppler echocardiography, Ro/SSA autoantibodies, home monitoring, systemic lupus erythematosus

## Abstract

**Objective:**

The aim of this study was to explore the parents’ experiences of home monitoring of the fetal heart rhythm. Women with anti-SSA/Ro52 autoantibodies carry a 2%–3% risk of giving birth to a child with congenital heart block (CHB), following transplacental transfer and antibody-mediated inflammation in the fetal conduction system during 18th to 24th gestational week. Early detection and subsequent treatment have been reported to decrease morbidity and mortality. Therefore, home monitoring of the fetal heart rhythm by Doppler has been offered at our fetal cardiology center. This study was undertaken to explore the lived experience of the routine.

**Methods:**

Participants were recruited from a single fetal cardiology center. Consecutive sampling was used. The inclusion criteria were women with SSA/Ro52 antibodies who had undergone Doppler examinations within the last two and a half years at the hospital and had monitored the fetal heartbeat at home. A semi-structured questionnaire was created, and the participants were interviewed individually. The interviews were transcribed verbatim and analyzed according to qualitative content analysis.

**Results:**

The overall theme was defined as “walking on thin ice,” with six underlying categories: reality, different strategies, gain and loss, healthcare providers, underlying tension, and conducting the examinations again, all with a focus on how to handle the home monitoring during the risk period.

**Conclusion:**

Both the mother and the co-parent expressed confidence in their own abilities and that the monitoring provided them with the advantage of growing a bond with the expected child. However, all the participants described a feeling of underlying tension during the risk period. The results show that home monitoring is not experienced as complicated or a burden for the parents-to-be and should be considered a vital part of the chain of care for mothers at risk for giving birth to a child with CHB. However, explaining the teamwork between the different caregivers, for the patients involved, their areas of expertise, and how they collaborate with the patient continues to be a pedagogic challenge and should be developed further.

## Introduction

For women with anti-SSA/Ro52 autoantibodies, the risk of giving birth to a child with congenital heart block (CHB) has been reported to be 2%–3%.^1–3^ The recurrence rate if the woman already gave birth to a child with CHB is reported to 12%–25%.^4–7^ The cause of this condition is antibody-mediated inflammation, which affects the atrioventricular node (AVN) in the fetus. More specifically, this reaction is mediated by maternal autoantibodies, of which the predominant types are anti-SSA/Ro52, associated with Sjögren’s syndrome and systemic lupus erythematosus (SLE), as well as women without other symptoms or signs of connective tissue disease. These antibodies can cross the placenta from the mother to the fetus, resulting in the observed inflammation in the AVN in susceptible fetuses and pregnancies.^8^ The heart block can be categorized into first to third degree atrioventricular (AV) block. A complete third-degree AV block is known to result in lifelong pacemaker treatment from early age and is the most severe manifestation of a fetal lupus syndrome.^4,5,9,10^ Without intervention, fetal death has been described in 15%–23% of affected pregnancies.^11–14^

When a heart block occurs, it has been described to progress between gestational weeks (GW) 18–24. Early signs of CHB can be detected using Doppler ultrasound echocardiography.^15,16^ The transition from a normal rhythm to a complete atrioventricular block can occur within less than 24 h. After early diagnosis of CHB, transplacental treatment with fluorinated steroids can be advocated, with the goal of limiting inflammation and reverting to a normal heart rhythm.^17^ Studies have suggested that treatment after the early detection of CHB decreases neonatal morbidity and overall mortality without increasing overall pregnancy complications.^18^ Reversal of an incomplete block, a ventricular escape rhythm of a higher rate, and the possibility of delaying pacemaker treatment to an older age have also been reported. Notably, there is no evidence of the positive effects of treatment in later stages during pregnancy.^19–21^ Hence, early detection of fetal heart blocks is crucial.

For anti-SSA/Ro52-positive women living with the threat of having a child with CHB, the pregnancy can be “put on hold” during the critical weeks (GW 18–24) due to worries for signs of CHB.^22^ The co-parents’ insight and experience are thus needed as they constitute an important support system for women with this condition.^23^

By enabling self-monitoring, the risk of CHB may be reduced through the possibility of early detection and treatment. Self-monitoring of the fetus has been shown to be safe and feasible.^24^ Women with various forms of high-risk pregnancies have generally had positive experiences when monitoring their fetuses at home by themselves.^25^ However, monitoring has to be performed alongside close interaction with healthcare professionals.^26^

Surveillance of the fetal heart rhythm with Doppler home monitoring has been performed in a few hospitals in Sweden. Accordingly, this study was conducted to explore how women, positive for anti-SSA/Ro52 autoantibodies and their co-parents experienced the home monitoring process.

## Aim

The aim of this study was to explore the expectant parents’ experiences of home monitoring of fetal heart rhythm.

## Methods

### Design

A qualitative research design was used. The reporting of the study followed the Consolidated criteria for reporting qualitative studies (COREQ).^27^

### Setting and participants

Participants were recruited with aid from healthcare professionals at a single fetal cardiology center in Stockholm, Sweden. A consecutive sampling was used, and all eligible women who fulfilled the inclusion criteria were invited to participate. The inclusion criteria included women with SSA/Ro52 antibodies who had undergone Doppler examinations during the last two and a half years at the hospital and who had monitored the fetal heartbeat at home. Exclusion criteria were non-Swedish or non-English-speaking women.

The eligible women (*n* = 29) were approached and received a letter with information about the study and a written consent form. Further, the women were asked to give an envelope with study information and a consent form to their co-parent if they had one. Women who did not respond received one reminder letter after 5 weeks. In all, 12 women and 6 co-parents gave their consent. However, one woman was excluded since she did not respond to the following contact (Table 1).

**Table 1.** Demographics of the participants.

Participants	Number, *n*	Mean (±SD)	Median (range)
Women*	11		
Age		37.3 (4.3)	
Rheumatic diagnosis			
Sjögren’s syndrome	4		
Systemic lupus erythematosus	1		
Undefined** (autoimmune disease and joint disease)	6		
Participating co-parents	6		
Age		39.3 (6.3)	
Number of months between the interviews and ending monitoring			16,5 (8–25)

*of 29 eligible women, response rate 38%, **described as a rheumatic condition by the participant.

### Data collection

Semi-structured individual interviews were carried out between September and November 2022. All interviews were conducted digitally, recorded, and lasted between 20 and 60 min.

Two separate interview guides were developed within the research group, one for the woman and one for the partner. The guides were developed with open-ended questions and aimed to focus on the research objectives. After asking general questions about the study participant, the interview moved on to more specific questions about the participant’s experiences with the monitoring process. The interviews were performed by the authors (JT, EÖ, and ÅB), and the interview guides were used to ensure alignment between the interviewers and the questions and topics discussed. Data saturation was obtained when replication of content was evident.

### Data analysis

The interviews were transcribed verbatim and analyzed using inductive content analysis, according to Elo and Kyngäs.^28^ In the present study, the analysis mainly focused on the manifest content of the transcribed text (i.e., what the text says). The content analysis entailed the identification of text units and open coding. The codes were grouped into categories that addressed the aim of the study. During the analysis, the researchers (EÖ, JT, and ÅB) compared the differences and similarities between their interpretations and a main category was then formed. To achieve trustworthiness, the authors (JT, EÖ, and ÅB) went back and forth in the text and compared and discussed the coding and categorization. Thematic saturation was obtained when replication of data or content was evident. From the resulting categories, an overall theme emerged that was considered representative of the emotional experiences of the families involved.

### Ethical considerations

All procedures were performed following the ethical standards of the institutional and/or national committee and with the 1964 Helsinki Declaration and comparable ethical standards. The study was approved by the Swedish Ethical Review Authority under the number Dnr 2022-01775-02.

## Results

All pregnancies, included in this study resulted in a living child, and none of the participants had a pregnancy in which the child developed CHB. However, two families stated that they had experienced a previous child with CHB.

**Figure 1.**
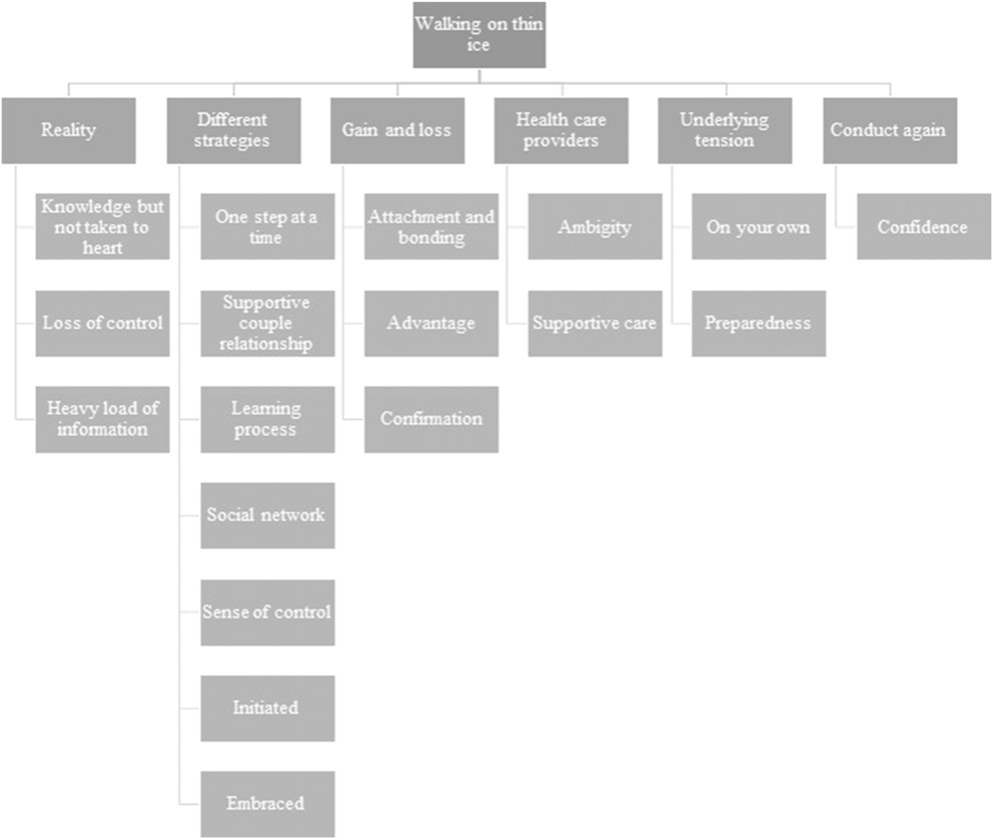
Theme and categories.

The overall theme was defined as “walking on thin ice,” with the underlying categories including “reality,” “different strategies,” “gain and loss,” “healthcare providers,” “underlying tension,” and “undergoing the examinations again” (Figure 1).

### Reality

The category of reality describes the awareness that women have about their potential risk for CHB. Information about the risk of CHB is given in connection with information on the diagnosis. However, the women and their partner often did not have a full understanding of the condition or the possible consequences. This led to an experience of being unprepared for what was to come once they were expecting a child and had to undergo surveillance with weekly controls at the cardiology clinic as well as daily home monitoring of the fetal heart rhythm. According to one participant, “We just got the referral, okay, we have an appointment, and so we go there at that time and day, but we didn’t know what it was or what to expect” (Co-parent 6). As another respondent indicated, “I knew that my wife had the autoantibodies and that there was a risk because we had been to the specialist together. But I do not think that I understood what it meant, what we had to do or the impact it had” (Co-parent 2).

For those participants with less insight about Ro/SSA autoantibodies and CHB, or for those that had not embraced their condition, the information given to them came as a shock. Aside from the perceived risk to the fetus, there was also a feeling of information overload. Since all families participated in the planned surveillance program, they had to visit the fetal cardiology unit weekly during the 18th to 24th gestational weeks. For those who lived farther away from the hospital, this required extensive planning within the family on the top of the burden of coping with this new vulnerable situation itself. According to one participant, “It was a bit overwhelming that we suddenly had to visit the cardiology clinic for the following six weeks. To get involved in the everyday puzzle… it became a huge, real, and serious thing…” (Mother 11).

When the risk of CHB had become a reality for the participants, some felt a loss of control, which was expressed as a fear of harming their unborn child and a state of shock, as well as a worsened experience of the pregnancy. From a medical perspective, while the fetus was not yet defined as a child, parents-to-be often referred to the fetus as their unborn child. Some participants also felt that the everyday situation was disorienting and experienced a feeling of being turned upside down. As one mother stated, “It came as a shock; everything had looked so good up until it didn’t” (Mother 2).

### Different strategies

When the participants had acclimatized themselves to the new situation involving home monitoring, they tackled it in different ways and found various strategies to feel sufficiently empowered to manage the situation. One strategy was to take one step at a time. They understood the seriousness of the responsibility they had and tried to prevent it from affecting everyday life in a negative way. Instead, they tried to construe the time spent checking their baby’s heartbeat as a positive situation, thereby strengthening the emotional connection between them.

A common approach among the couples was to do the checks together. By being together in a partnership, they could give support to each other and interpret the heart sounds together. Some partners expressed a sense of security in being able to monitor the fetal heart rhythm at home. As one mother indicated, “My partner didn’t know much, so I thought it was good for him to join me in this and to ask questions if he wanted. That is how we reasoned through the process” (Mother 8).

Learning how to find and interpret sounds was another complicated process for the participants. However, after some practice, it became an everyday routine. The security of hearing the heart sounds became an incentive to do the checks. As one mother reported, “After one week or two, you were into it, and it became nice to do the checks and be sure that the heart was ticking…” (Mother 10). Generally, the participants who had undergone such check-ups in a previous pregnancy felt more confident in their own abilities. For one mother, “With the second child, it was more like, ‘Have we checked? Oops, we have forgotten’, but then we do it now. It was totally different [the second time]. Now you were an expert on this thing…” (Mother 1).

In this study, the majority of the participants felt well informed about the risks associated with their condition. They also felt that the healthcare professionals at the cardiology clinic were experienced and competent. In one mother’s view, “I think we got a good strategy from the healthcare team that helped us, and I believe it was due to their experience in the area. They gave us a lot of information. It was really good, and it felt like a luxury to take part in this, given the circumstances” (Mother 3).

Among the participants, there was a variation in whether they chose to share the situation they were experiencing with friends or relatives. Some wanted to keep it a secret, while others sought security and support in their social contacts. For some participants, despite good support and advice, there was still a need for affirmation and comfort: “I feel that I am the kind of person who would like any adult, within the healthcare profession, to hug me and say that it will be all right” (Mother 1).

### Gain and loss

There was an overall positive effect of home monitoring, due to the current context, which created an opportunity for the participants to establish a moment of daily contact with their baby. As one partner reported, as a result of this process, “there was a lot of communication with the baby” (Co-parent 6).

Home monitoring generated a feeling of advantage for some participants, given that it allowed for continuity in contact with the baby and a routine in which the participants continuously confirmed that the condition was good. Even though there was a bit of tension until they could hear the heartbeat. None of the participants stated that home monitoring was a burden and instead interpreted it with a sense of privilege. As one mother indicated, “We listened every day, and we made it like a routine. It became a cosy moment when we listened to the heartbeat; it was not with fear, but more like a nice feeling” (Mother 6).

With the possibility of home monitoring, the participants felt a safe confirmation about their baby’s health, which made everyday life work more efficiently. Even though it was difficult for some participants to directly locate the baby’s heartbeat, the overall feeling was a confirmatory one that relieved stress. According to one mother, “I thought it was stressful to do it before work, but in the afternoon, when coming home, or in the evening, I felt calmer. Yes, then you could go to sleep with a clear conscience” (Mother 10).

### Healthcare providers

The participants experienced, in several cases, that maternity care showed a lack of knowledge about rheumatic diseases. Even though specialists were involved, there was still a sense of ambiguity experienced by the pregnant mothers about their responsibilities. Specifically, there was a feeling among the mothers that there was a lack of communication and an unclear differentiation of roles between the different healthcare providers. One partner reported that “it was a bit messy. My partner had a midwife and a rheumatologist watching her (the baby). Sometimes, we did tests in one hospital, and sometimes in another; it was different doctors every time. There was a feeling that things could get out of hand, given the triangle of healthcare givers” (Co-parent 1).

Despite the stressful situation for the participants, most felt that the specialists at the cardiology clinic were supportive, gave adequate information, and established a sense of trust. The participants also felt confidence and capable of performing the home monitoring with the instructions and support they received from the cardiology unit. As one mother revealed, “One was definitely not left alone. And they also asked me if I remembered correctly every week and asked how things were going” (Mother 9).

### Underlying tension

After the period of home monitoring was concluded, there were different experiences and levels of contact with the cardiology clinic. Some participants felt it was like a natural ending of something and that the critical period was over. For some, this was like a relief. One co-parent reported, “We had to return the device, and then we had no opportunity to do it anymore. It was a natural ending” (Co-parent 2).

Other participants felt that they had been abandoned after home monitoring. The close relationship with the specialists ceased, and they had a feeling of being solely responsible for scheduling any follow-ups after birth. As one mother indicated, “My feeling was that if we didn’t grab the opportunity to book an appointment after birth, there was the feeling that it was on us to do that. There was no feeling that there were any other follow-ups on us” (Mother 6).

Among some participants, feelings of increased responsibility, guilt, and worry emerged during their pregnancy. The guilt of being pregnant despite the risk and carrying the responsibility of managing the home monitoring process led to fear and anxiety regarding what could happen to the baby. For one mother, simply put, “It felt like a big responsibility” (Mother 2). Even though the home monitoring process itself gave the participants a sense of control and empowerment, as described above, many experienced an underlying tension and prepared themselves for something to go wrong: “You were calm because you had the device, but you still worried all the time that you might find something…” (Co-parent 1).

### Conduct home monitoring surveillance again

The overall experience of home monitoring was that it was easy to manage and gave the parents a feeling of confidence in this special situation. According to one mother in particular, “If there will be more pregnancies, I would gladly do it again, as I would feel more security” (Mother 10). Generally, the participants who had undergone home monitoring before were willing to conduct it again.

## Discussion

This interview study was conducted to better understand the experience of home monitoring of fetal heart rhythm for the families involved. Given that CHB can develop in less than 24 h, and with the possibility of preventing the condition through early treatment,^17,24^ daily surveillance is crucial for early detection. When daily monitoring, there is a need to support expecting parents to carry out the process by themselves at home. In the present study, we found that the home monitoring procedure was generally well tolerated by the women and their co-parents.

In other studies, home monitoring has been shown to be acceptable without posing an excess burden or causing unnecessary concern.^29^ It should be noted that in all home monitoring programs, there has always been a responsible caregiver who could be contacted in the case of any signs of abnormality. In this study, the expecting parents stated that they were willing to conduct home monitoring again in future pregnancies, and this experience was also reported by others.^24^

Fetal echocardiography has also been suggested to have a positive impact on maternal–fetal attachment.^30^ Prenatal attachment can influence good health practices during pregnancy and facilitate adaptation to the role of parenthood.^31^ Several factors affect prenatal bonding, such as maternal and paternal emotional states.^32^ For parents who learn that they are expecting a child with a heart defect, life is affected both during and after pregnancy and can have a negative impact on early attachment to the expected child and the transition to parenthood.^33,34^ When conducting home monitoring, the participants in this study confirmed that hearing the baby’s heartbeat established a moment of connection with their baby and a confirmation that their condition was good.

Even though home monitoring has its benefits, there are several parameters that must be managed before its implementation. Prenatal diagnoses of congenital heart decease (CHD) have been demonstrated to generate maternal stress, which can lead to the production of elevated maternal cortisol levels.^35^ Bratt et al. showed that parents who found out about their offspring’s CHD showed poorer life satisfaction and a limited sense of coherence than a healthy control group.^36^ In the present study, the parents-to-be indicated that they experienced anxiety after receiving information about the potential risk of CHB. The anxiety in relation to the heavy information load made the parents feel as if they had entered a vacuum, replete with a state of confusion and tunnel vision. Regardless, the healthcare staff have a vital role to engage with the parents and ensure that they have taken in all the relevant information, including making sure to follow-up on reactions that might evolve.

In this study, the interviewed mothers and co-parents had a strong sense of trust in the healthcare system. However, it should be noted that this was essentially based on their confidence in the healthcare personnel at the cardiology clinic. It has been shown before that expecting parents have reported a significant lack of clarity about where their responsibilities lay.^22^ Some participants in this study described feeling alone after the surveillance period, which was also noted in a previous study in which women expressed a feeling of being in a vacuum after regular visits for fetal echocardiography.^37^

Furthermore, the participants had different feelings regarding the involvement of their social networks. Some chose to keep the situation to themselves, while others chose to share it with their families and friends. In a recent study conducted in 2023, interviewed women with a high-risk pregnancy stated that they were building “nesting networks” to support them through pregnancy.^38^ The women also reported that their midwives were their first point of contact. Generally, women who knew that they could contact their midwives felt supported. In other studies, pregnant women with anti-SSA/Ro52 have reported that maternity care in general shows a lack of knowledge about rheumatic diseases and related risks,^22,39^ which is similar to the findings of this study.

However, to explain the teamwork between the different caregivers, for the patient involved, and their areas of expertise and how they collaborate around the patient and the situation continues to be a pedagogic challenge. The cardiologists have a responsibility to clarify their own role in relation to other caregivers. Supporting effective teamwork between the different caregivers, improvement in their areas of expertise, and the promotion of strong collaboration with a patient need to be improved.

### Limitations/considerations

A limitation of this study was that we did not collect data on the participants’ socioeconomic status, which could have impacted how they reacted to and processed the information. It should also be noted that for all participants, there was some time between the conclusion of home monitoring and the interviews. This might have affected their memory of different time points and of the process itself. However, the overall feeling of being at risk and listening to the fetal heart to detect discrepancies during pregnancy was well described by the women and their partners. Furthermore, during 2020–2022, the COVID-19 pandemic was persistent, which meant restrictions for relatives visiting the hospitals and taking part in the Doppler examinations. This, in turn, might have affected the experience.

There was also a limitation regarding the fact that not all the people invited to share their experiences agreed to participate. Also, fewer co-parents/partners than mothers agreed to participate. For various reasons, it would be valuable to determine why a person agrees to participate.

## Conclusions

Being informed about the risks of CHB was experienced as a stressful situation for the majority of participants. Home monitoring seems to strengthen the expectant parents to take on the responsibility of handling daily surveillance. The respondents reported that life in general continued to be normal and that surveillance was a relief, providing a sense of security in the provision of daily control. Both the expecting mother and the co-parent expressed confidence in their abilities and reported that the monitoring provided them with the advantage of growing a bond with the expected child. However, all the participants described a feeling of underlying tension during the risk period. Home monitoring was not perceived as a problem that would affect future pregnancies among the interviewed parents. The attitude was that they were willing to conduct home monitoring again if they chose to become pregnant at a later time. The results of this study show that home monitoring contributes to a feeling of confidence and should be considered a vital part of the chain of care for mothers at risk of giving birth to a child with CHB.

## Clinical implication and next step

The result of this study indicates that home monitoring of heart sounds can contribute as an important complement to medical follow-ups. This study was conducted in a highly specialized single center, with a program of weekly monitoring of fetal AV time intervals, which may affect the generalizability. Although, our opinion is that pregnancies, with known maternal anti-SSA/Ro52 autoantibodies, outside areas with highly specialized fetal cardiology programs would benefit even more from the described home monitoring, with the potential of earlier detection of CHB. One could argue for incorporation of home monitoring into basic programs of maternal care in case of maternal anti-SSA/Ro52 autoantibodies. Further research is needed to investigate the outcomes and experiences of a population in a wider setting.
